# HER3 (ERBB3) amplification in liposarcoma - a putative new therapeutic target?

**DOI:** 10.1186/s12957-024-03406-5

**Published:** 2024-05-17

**Authors:** Ann-Katharina Becker, Behrus Puladi, Kunpeng Xie, Angela Cassataro, Rebekka Götzl, Frank Hölzle, Justus P. Beier, Ruth Knüchel-Clarke, Till Braunschweig

**Affiliations:** 1 Institute of Pathology, University Hospital RWTH Aachen, 52074 Aachen, Germany; 2 Department of Oral and Maxillofacial Surgery, University Hospital RWTH Aachen, 52074 Aachen, Germany; 3 Department of Plastic, Hand Surgery – Burn Center, University Hospital RWTH Aachen, 52074 Aachen, Germany; 4 Institute of Pathology, Faculty of Medicine, LMU Munich, Thalkirchner Strasse 36, 80337 Munich, Germany; 5 Center for Integrated Oncology Aachen Bonn Cologne Duesseldorf (CIO ABCD), Aachen, Germany

**Keywords:** Liposarcoma, HER3, ERBB3, Coamplification, MDM2 amplification, DDIT3, Fluoresence in situ hybridization (FISH) immunohistochemistry

## Abstract

**Background:**

Liposarcomas are among the most common mesenchymal malignancies. However, the therapeutic options are still very limited and so far, targeted therapies had not yet been established. Immunotherapy, which has been a breakthrough in other oncological entities, seems to have no efficacy in liposarcoma. Complicating matters further, classification remains difficult due to the diversity of morphologies and nonspecific or absent markers in immunohistochemistry, leaving molecular pathology using FISH or sequencing as best options. Many liposarcomas harbor MDM2 gene amplifications. In close relation to the gene locus of MDM2, HER3 (ERBB3) gene is present and co-amplification could occur. Since the group of HER/EGFR receptor tyrosine kinases and its inhibitors/antibodies play a role in a broad spectrum of oncological diseases and treatments, and some HER3 inhibitors/antibodies are already under clinical investigation, we hypothesized that in case of HER3 co-amplifications a tumor might bear a further potential therapeutic target.

**Methods:**

We performed FISH analysis (MDM2, DDIT3, HER3) in 56 archived cases and subsequently performed reclassification to confirm the diagnosis of liposarcoma.

**Results:**

Next to 16 out of 56 cases needed to be re-classified, in 20 out of 54 cases, a cluster-amplification of HER3 could be detected, significantly correlating with MDM2 amplification. Our study shows that the entity of liposarcomas show specific molecular characteristics leading to reclassify archived cases by modern, established methodologies. Additionally, in 57.1% of these cases, HER3 was cluster-amplified profusely, presenting a putative therapeutic target for targeted therapy.

**Conclusion:**

Our study serves as the initial basis for further investigation of the HER3 gene as a putative therapeutic target in liposarcoma.

## Introduction

Liposarcomas (LPS) are among the most common entities of malignant mesenchymal tumors, accounting for about 5 to 15% of all adult soft tissue sarcomas (STS) [1–3] and are morphologically divided into four main subgroups: (1) atypical lipomatous tumors (ALT)/well-differentiated LPS (WDLS); further collectively referred to well differentiated liposarcoma - WDLS, (2) dedifferentiated LPS (DDLS), (3) myxoid or round cell LPS (MLS) and (4) pleomorphic LPS (PLS). Recently, a fifth, very rare subtype in young individuals was defined by the 5th edition of WHO Classification of soft tissue tumors [4], the myxoid pleomorphic liposarcoma with mixed morphology [5, 6].

Based on genetic alterations being specific and exclusive for most cases of several subgroups, the first two subgroups can be merged: well-differentiated and dedifferentiated LPS form a common group due to their similar genetics, showing characteristically so-called ring or giant marker chromosomes [7, 8]. Furthermore, both types of LPS have an amplification of chromosome 12q13-15, which include well described gene amplifications used as molecular diagnostic markers (MDM2, CDK4) as well as others in recent studies (e.g. HMGA2, CPM, CDKN2A) [7–10]. In contrast, myxoid liposarcomas are characterized in 90–95% of cases by a translocation t(12;16)(q13;p11) and the resulting fusion gene FUS-DDIT3 (CHOP) and its translated fusion oncoprotein, utilized also in diagnosis [11, 12]. The fourth group of LPS are pleomorphic liposarcomas (PLS) harbor complex and not uniform genetic changes, caused by loss of function of tumor suppressor genes p53 and RB1 (retinoblastoma) among others, but can be diagnosed by histologically characteristic pleomorphic nuclei and high proliferation [13]. The last group of LPS, myxoid pleomorphic LPS shows generally chromosomal loss in 13q14 and a monoallelic RB1 deletion could be observed [6]. Next to specific genetic alterations, the different subtypes occur in different body compartments, as subcutaneous tissue of the extremities, thorax (e.g., WDLS) or retroperitoneum (e.g., MLS, DDLS). The fifth subgroup is often found in the mediastinum [14].

As genetic background of subtypes of LPS is different, so is prognosis [15]. While well-differentiated liposarcomas have a negligible risk for metastatic potential [16], dedifferentiated liposarcomas have a less favorable prognosis due to a local recurrence rate of 41%, a metastatic rate of 17%, and a disease-related mortality rate of 28% [17]. As retrospective studies included genetic alterations in the analysis of survival data, general co-amplifications and other mutations showed associations with worse outcome [18], while others looked specifically on co-amplifications of receptor tyrosine kinases (RTK) and could not find any significant difference in survival [10]. In myxoid LPS, the proportion of round cell component should be considered [19, 20]. Among all liposarcomas, pleomorphic liposarcomas have the worst prognosis [21, 22] with a reported local recurrence risk of 34–45% as well as a risk of metastasis of about 32–57% [23].

As RTK play a crucial role in cell cycle and differentiation, they are used as therapeutical targets in a broad spectrum of oncological diseases [24]. Next to a blockade of RTKs using monoclonal antibodies, specific inhibitors were developed. In recent years, conjugates of antibodies and cytostatic drugs were developed and tested successfully [25]. In breast carcinoma a conjugate is already approved by the FDA and EMA [26]. There are 20 classes of RTKs of which the first sums the EGF receptor resp. HER (ERB) subtypes. That subgroup includes four receptors with a similar structure. Yet, EGFR and HER2 are target structures in several different cancer types and a huge variety of drugs [24]. Concerning the other two subtypes, HER3 (ERBB3) and HER4, inhibitors are under investigation in several clinical trials as targets in precision medicine of malignant tumors [27]. Several so called pan-inhibitors, targeting several subtypes of this group are mentioned in publications [9, 28].

Due to spatial close relation to the MDM2 locus, the presented study is focused on the copy number analysis and expression of the HER3 receptor in LPS. The HER3 gene is located on the long arm of chromosome 12 (position 12q13). Thus, it is in close range to MDM2 at position 12q13-14 and CDK4 at position 12q14. Even though some publications refer to co-amplified RTK in WDLS and DDLS, few were described as rare as DDR2, NTRK1 and even less frequent HER3 [10].

The aim of this study is to characterize the amplification of HER3 in archived cases of LPS using FISH and immunohistochemistry. We hypothesize that this information may provide the rationale for using HER3 amplification for more accurate classification and/or as a potential therapeutic target.

## Materials and methods

A total of 56 cases of lipomatous tumors were available as sufficient formalin fixed, paraffin embedded samples. The study was approved as a retrospective, anonymized study by the ethical commission of the University Hospital RWTH Aachen, Germany (approval no. EK 176/06). As samples are fully anonymized, a written consent by patients is not applicable. Inclusion criteria were next to diagnosis that samples were not biopsies, but resections and representative and homogeneous tumor was available.

### Investigation

**Fig. 1:**
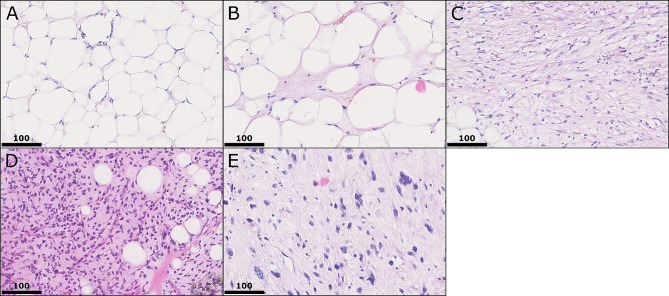
Histological subtypes. Overview of typical histology of included lipomatous neoplasms. In (**A)** benign lipoma, consisting of homogeneously shaped lipocytes with slim nuclei at cellular borders; in (**B)** well differentiated liposarcoma shows atypical lipocytes with different, inhomogeneous diameters and septa with slight nuclear atypia with triangular shaped nulcei with pointed edges. In (**C)** dedifferentiated liposarcoma is seen with some small tumor cells with vacuoles and spindle shaped tumor cells in matrix rich stroma and up to intermediate nuclear atypia. In (**D)** myxoid liposarcoma is shown with typical myxoid stroma and densely grown tumor cells with triangular nuclei. In (**E)**, characterized by severe pleomorphic and atypical nuclei, pleomorphic liposarcoma is seen in matrix rich stroma with possible single tumor cells with residual vacuoles. All stains hematoxylin and eosin, all images by 400x magnification, and scale bar equals 100 μm

Samples were reviewed by sections stained by hematoxylin and eosin (H&E) and tumor areas were detected (Fig. 1). Tissue microarrays (TMA) were constructed using a semiautomated tissue arrayer and 3 representative cores of each case were included (Pathology Devices, San Diego, CA, USA). 5 μm sections of the TMAs were stained by standard H&E and immunohistochemistry. Furthermore, fluorescence in-situ hybridization (FISH) was performed. The TMAs were evaluated first by H&E to confirm the content in respect to the original diagnosis and tumor tissue.

**Fig. 2:**
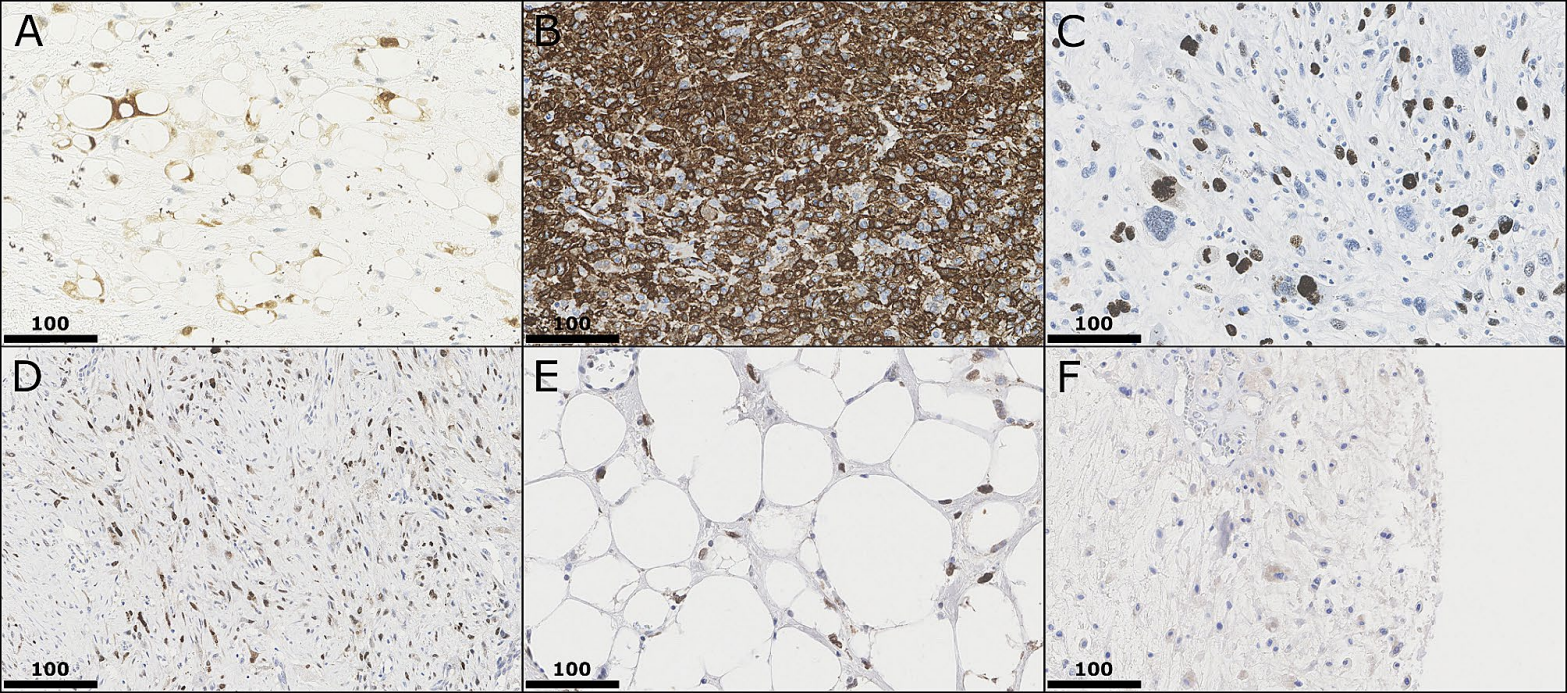
Immunohistochemistry in different subtypes. In (**A**) stain for S100 showing nuclear and cytoplasmic positivity in a dedifferentiated liposarcoma. In (**B**) a myxoid liposarcoma shows a broad positive stain in vimentin; Ki67 is demonstrated in (**C**) in a pleomorphic liposarcoma with nuclear stain in mitotic active tumor cells. In (**D**) a dedifferentiated liposarcoma shows positive nuclear stain for MDM2, in (**E**) a well differentiated liposarcoma is positively stained for CDK4. In (**F**) HER3 is slightly positive in a subset of tumor cells in a tumor with mixed morphology. All images by 400x magnification and scale bar equals 100 μm

Immunohistochemistry was done by using Flex Kit by Agilent/Dako (Agilent, Santa Clara, USA) and autostainer 360 (ThermoFisher Scientific, Waltham, USA). TMAs were stained by immunohistochemistry with antibodies for Ki67 (MIB-1, Agilent; RRID: AB_2631211), S100 (polyclonal, Agilent; RRID: AB_2811056), MDM2 (IF2, Life Technologies, Carlsbad, CA, USA; RRID: AB_2533136), CDK4 (DCS-31, Invitrogen, Carlsbad, CA; USA; RRID: AB_667450), vimentin (V9, Agilent; RRID: AB_10013485) and HER3 (Fig. 2). For HER3, we tested two different clones: 2F12 (Merck, Darmstadt, Germany; RRID: AB_11211839) and D22C (Cell Signaling Technology, Cambridge, UK; RRID: AB_2799907). Both antibodies didn’t perform as specific as expected but we decided to use D22C in this study for its slightly better characteristics.

H&E and immunohistochemistry stains were evaluated by digitalized slides, using a whole slide scanning device (Hamamatsu, NanoZoomer, 2.0-HT, Hamamatsu, Hamamatsu City, Japan) and viewing software ndpview (Hamamatsu).

**Fig. 3:**
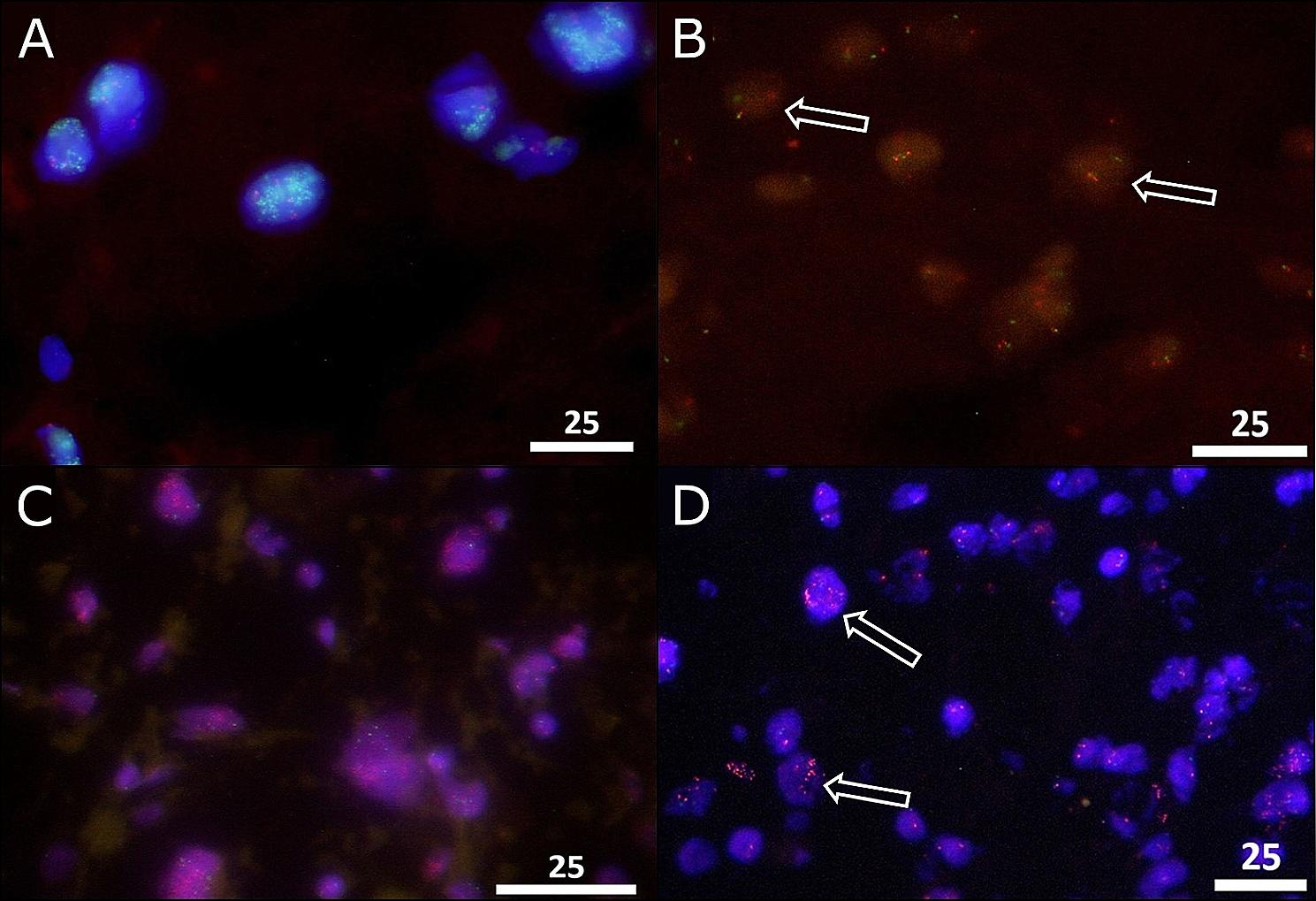
Fluorescence in-situ-hybridization. Examples of applied probes for the detection of amplifications or rearrangement. In (**A**) the typical MDM2 cluster-amplification in a dedifferentiated liposarcoma in all tumor cells with accumulated, cloud-like green signals next to two red centromere signals. In (**B**) break-apart probes for (FUS-)DDIT3 show in case of rearrangement next to overlaying or close green/red signals two separate green/red signals within the nucleus (see arrows) in a myxoid liposarcoma. In (**C**), a broad cluster-amplification of HER3 with cloud-like accumulated red signals in all tumor cells is visible in a dedifferentiated liposarcoma next to two green centromere signals; in (**D**) HER3 cluster-amplification was detected in a subset of tumor cells (arrows). All images by 1000x magnification, and scale bar equals 25 μm

MDM2 amplification as well as HER3 co-amplification and DDIT3 translocation were examined by fluorescence-in-situ hybridization (FISH) using FISH Accessory Kit (Z2028), probes for the target genes (SPEC-MDM2(Z2013), SPEC-DDIT3(Z-2100), SPEC-ERBB3(Z2056))) and DAPI (MT-008) for nuclear counterstain (all by Zytovision, Zytomed Systems, Berlin, Germany) (Fig. 3). Hybridization was performed on ThermoBrite hybridizing system (Abbott Molecular, Chicago, USA). Signals were made visible on a Zeiss invers microscope, Axiovert 135 (Zeiss, Oberkochem, Germany) and specific filters (AHF Analyse Technik, Tubingen, Germany). Microphotographs were achieved by a CCD camera (KY-F75U, JVC, Yokohama, Japan) and DISKUS software (Hilgers, Königswinter, Germany).

### Evaluation

On stained slides of TMAs, immunohistochemistry was scored by using the immunoreactivity score (IRS) a product of scores of intensities of stain (0–3: 0 = no color reaction, 1 = mild reaction, 2 = moderate reaction, 3 = intense reaction) and stained cells (0–4: 0 = no positive cells, 1 = < 10% positive cells, 2 = 10–50% positive cells, 3 = 51–80% positive cells, 4 = > 80% positive cells). Scores were categorized by negative {0–2}, low positive {3–4}, intermedium positive {6–8} and strong positive {9–12}, scores of 3 up to 12 were counted as a positive stain.

Interpretations of signals was different for amplification (MDM2, HER3) and translocation/break apart (DDIT3). For amplification a ratio of gene and centromere signals of ≥ 2.0 counting 60 tumor cells was considered amplified. In case of cluster amplifications (cloud of numerous gene signals), tumors with > 30 cells with cluster amplification were considered highly cluster-amplified, cases with < 5 cells with cluster-amplification were named focally amplified. In case of FUS-DDIT3 50 cells were counted. In case of > 15% cells with break apart signals (two separate-colored signals instead of an overlayed color signal), cases were categorized as positive (rearranged).

### Statistical analysis

R programming language was used for statistics (Fishers Exact Test). A *p*-value < 0.05 was considered statistically significant.

## Results

Samples were distributed by age between 21 and 80y, only one below 40y, mostly between 41 and 60y (22 cases, 39%) and between 61 and 80y (27 cases, 48%) and much less over 80 years (6 cases, 10%). Concerning gender-distribution, we found higher rates of male patients in DDLS, PLS and MLS and in WDLS higher rates in female.

Archived cases were sub-grouped by their original diagnosis and therefore stratified into the four main subtypes, next to a control group of benign lipomas and a mixed subgroup, presenting an inhomogeneous (immune-)histological pattern but the original diagnosis of liposarcoma. Samples are summarized in Table 1.

**Table 1:** Histomorphological subtyping of samples of included lipomatous tumors

Type	WDLS	DDLS	MLS	PLS	Mixed LPS	Lipoma (Control)
Number	17 (29%)	19 (34%)	7 (12%)	5 (9%)	3 (5%)	5 (9%)
Gender	f > m	m > > f	m > > f	m > f	m > f	m > f

In immunohistochemistry (IHC) (Table 2), vimentin was positively stained in all cases while S100 was inhomogeneously stained with significant positivity in WDLS and DDLS. Higher amounts of Ki67 expression could be observed in single cases of myxoid and in pleomorphic liposarcomas. Expression of MDM2 by IHC was inhomogeneously positive in all subgroups. Within the mixed subgroups 1 of 3 was positive (33.3%) and none of the included control group of lipomas. CDK4 expression correlated significantly with MDM2. All MDM2 positive tumors co-expressed CDK4.

**Table 2:** Immunohistochemistry on all archival subgroups of cases

Type	Total No. Of Cases	Immunohistochemistry (IHC)				
		Vimentin	S100	MDM2	CDK4	HER3
Lipoma (Control)	5	5 (100%)	5 (100%)	0 (0%)	0 (0%)	0 (0%)
WDLS	17	16 (98.2%)	12 (70.6%)	8 (47.1%)	8 (47.1%)	5 (29.4%)
DDLS	19	19 (100%)	8 (42.1%)	15 (78.9%)	15 (78.9%)	7 (36.8%)
MLS	7	7 (100%)	5 (71.4%)	3 (42.9%)	3 (42.9%)	2 (28.6%)
PLS	5	5 (100%)	4 (80%)	3 (60%)	4 (80%)	2 (40%)
Mixed LPS	3	3 (100%)	3 (100%)	1 (33.3%)	2 (66.7%)	1 (33.3%)
Total (w/o controls)	51	50 (98.0%)	32 (62.7%)	30 (58.8%)	32 (62.7%)	17 (33.3%)

By immunohistochemistry, positively stained cases are summed up in percentages of tumor subgroups by their original diagnoses. Vimentin, as a general marker of mesenchymal origin was positive in basically all cases, proving the origin. S100, said to be specific in WDLS, DDLS and less positive or negative in MLS and PLS, shows inhomogeneous stain throughout all cases. MDM2 and CDK4 are significantly correlated and show as well as significant correlation to MDM2 amplification in FISH diagnostic. HER3 was low to intermedium stained in cases HER3 was amplified and demonstrates a rather cytoplasmic staining

Results of FISH analysis are summarized in Table 3. Generally, in case of amplification, only cluster-amplifications were observed. There was no amplification by numeric, ratio-related amplification visible.

**Table 3:** FISH on all subgroups of archived cases

Type	Total No. Of Cases	Fluorescence In-Situ-Hybridization (FISH)				
		MDM2	DDIT3	HER3+ (Focal)	HER3 (Broad)	HER3 (Total)
Lipoma (Control)	5	0 (0%)	0 (0%)	0 (0%)	0 (0%)	0 (0%)
WDLS	17	11 (64.7%)	0 (0%)	4 (23.5%)	2 (11.8%)	6 (35.3%)
DDLS	19	15 (78.9%)	0 (0%)	4 (21.1%)	5 (26.3%)	9 (47.4%)
MLS	7	3 (42.9%)	2 (28.6%)	2 (28.6%)	0 (0%)	2 (28.6%)
PLS	5	3 (60%)	0 (0%)	0 (0%)	2 (40%)	2 (40%)
Mixed LPS	3	1 (33.3%)	0 (0%)	0 (0%)	1 (33.3%)	1 (33.3%)
Total (w/o controls)	51	33 (58.9%)	2 (3.6%)	10 (17.9%)	10 (17.9%)	20 (39.2%)

Analysis with MDM2 probe demonstrates over half of the cases cluster-amplified in well differentiated and dedifferentiated liposarcoma, as well as in the class of pleomorphic tumors and less in myxoid appearing liposarcomas and liposarcomas of mixed histological subtypes thus leading to reassignments unless the amplification of MDM2 is defined as specific for the subgroups of well differentiated and dedifferentiated liposarcomas. DDIT3 rearrangements were observed in only two cases with myxoid histomorphology, proving a myxoid liposarcoma

By FISH analysis of gene locus MDM2, 59% (33 cases) of the samples showed an amplification of MDM2, most cases within the WDLS/DDLS subgroup, matching significantly to IHC. Almost half of the MLS and PLS cases and one of the three mixed tumors were MDM2 amplified as well (33%). No amplification was found in lipomas. Within the control group, no positive staining or amplification of MDM2 could be found.

By FISH analysis for break apart probes of DDIT3, two of the primarily as myxoid liposarcomas classified cases showed a rearrangement signal.

FISH analysis of HER3 showed cluster-amplifications in broad areas (ba) or focally (f), present in more than half but not all cases with MDM2 cluster-amplifications. In WDLS, 6 of 11 MDM2 amplified cases were positive (2ba/4f), in DDLS, 9 of 15 MDM2 amplified samples showed HER3 amplifications (5ba/4f) whereas in MLS, two of three MDM2 amplified cases showed small areas of HER3 cluster-amplifications. Two of three PLS showed -as the single case within the mixed subgroup with MDM2 amplification- broad areas of HER3 cluster-amplification. In total, 10 (broad) or 10 (focal) of 33 cases presented with co-amplifications of HER3 and MDM2, highly correlated (*p* < 0.001 and *p* < 0.05, respectively, Fisher’s Exact Test). There was no sample identified with HER3 amplification without MDM2 amplification, indicating a very strong overall co-occurrence.

**Fig. 4:**
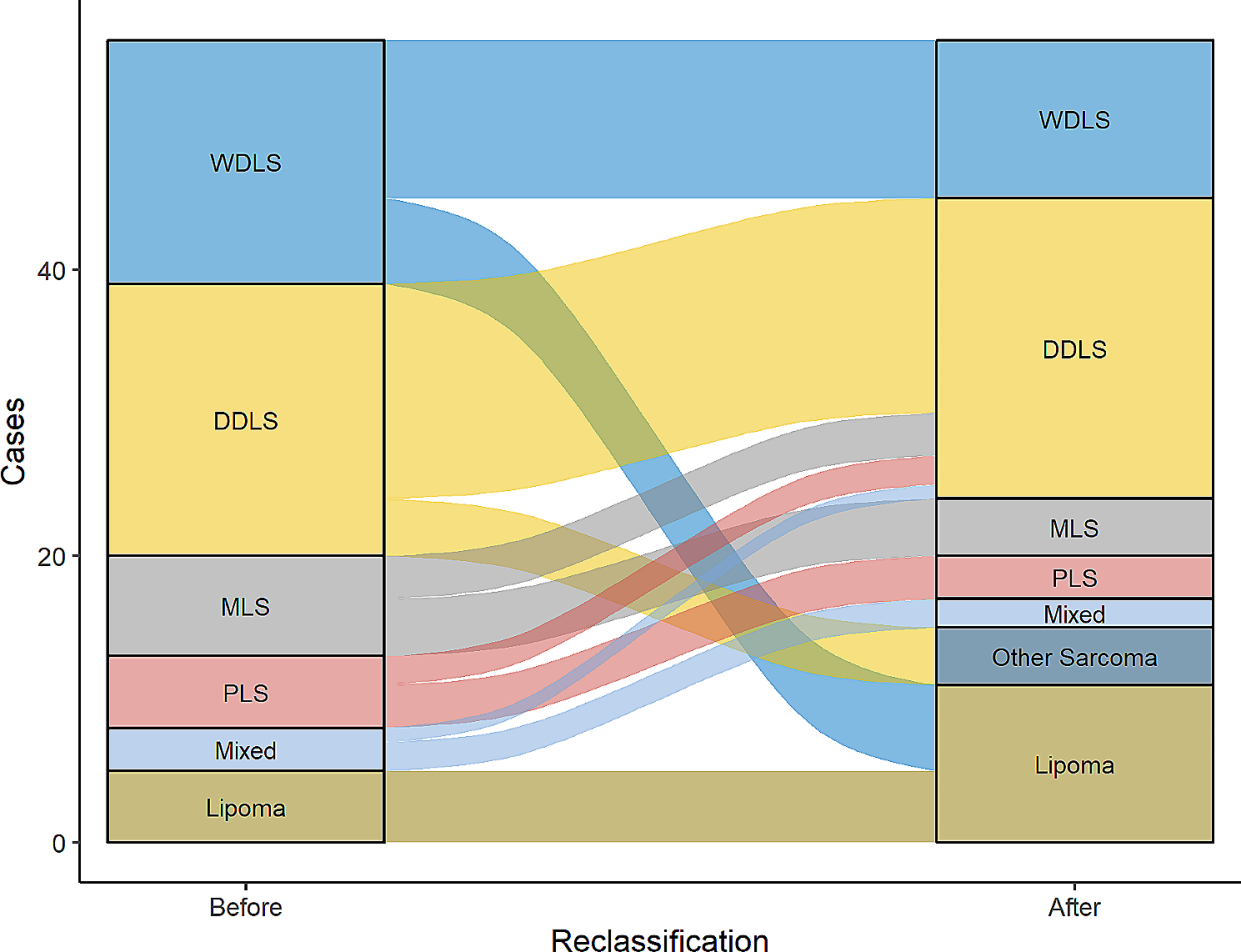
Reclassification of tumors. In conclusion of immunohistochemistry and fluorescence-in-situ-hybridization, a total of 17 cases needed to be reassigned. Most cases changed subtype in the group of well differentiated liposarcomas by not showing MDM2 amplification and therefor were placed to lipomas. Within the subgroup of dedifferentiated liposarcomas, 4 cases out of 19 were not MDM2 amplified and needed to be reassigned to unknown or other sarcoma subgroup. Cases within MLS, PLS and the group of mixed histology with MDM2 amplification were included in the dedifferentiated liposarcoma subgroup

As a side-effect, by immunohistochemistry and FISH results, cases needed to be reclassified (Fig. 4; Table 4). As a result, only 35 of the 51 cases were proven to be liposarcomas. In 20 of 35 (57.1%) of these cases, HER3 amplification was present (Table 5).

**Table 4:** Diagnostic algorithm of liposarcomas by immunohistochemistry and FISH

Criteria	Method	WDLS	DDLS	MLS	PLS	Lipoma
Morphology	H&E	atypical lipocytes with different, inhomogeneous diameters and septa with slight nuclear atypia with triangular shape and sharp edges	spindle shaped tumor cells in matrix rich stroma and up to intermediate nuclear atypia, some or areas of atypical vacuolated lipocytes	typical myxoid stroma and relatively close packed, triangular nuclei of tumor cells	severe pleomorphic and atypical nuclei of tumor cells in matrix rich stroma with possible single tumor cells with residual vacuoles	homogeneously shaped, adult lipocytes with slim nuclei at cellular borders
Immunohistochemistry	S100	**+**	**+**	**+**	**+/-**	**+**
	Vimentin	**+**	**+**	**+**	**+**	**+**
	MDM2	**+**	**+**	**-**	**-**	**-**
	CDK2	**+**	**+**	**-**	**-**	**-**
	Ki67	**low**	**(+)**	**+**	**+++**	**-**
FISH	MDM2	**+**	**+**	**-**	**-**	**-**
	DDIT3	**-**	**-**	**+**	**-**	**-**
	HER3	**+/-**	**+/-**	**+/-**	**+/-**	**-**

**Table 5:** HER3 in the reclassified cases

Type	Total No. Of Cases	HER3 (FISH)		
		Focal	Broad	Total
WDLS	11	4 (36.4%)	2 (18.2%)	6 (54.5%)
DDLS	15	4 (26.7%)	5 (33.3%)	9 (60%)
MLS	5	2 (40%)	0 (0%)	2 (40%)
PLS	3	0 (0%)	2 (66.7%)	2 (66.7%)
Mixed LPS	1	0 (0%)	1 (100%)	1 (100%)
Total	35	10 (28.6%)	10 (28.6%)	20 (57.1%)

## Discussion

Differential diagnosis of liposarcomas remains challenging. A combination of localization, morphology, immunohistochemistry and genetic investigation is recommended and necessary [29]. As immunohistochemistry is not specific in marker constellation (S100, MDM2, CDK4) for subtypes of liposarcomas but in groups [8, 30] the establishment of fluorescence-in-situ hybridization for diagnostic stratification (around 2013) paved the way to define singles entities.

HER3 amplifications were found in 20 cases, representing 39.2% (20/51 cases) of primarily included liposarcomas (Table 3) and 57.1% (20/35) of the reclassified cases (Table 5). In half of HER3 amplified cases, MDM2 amplification and HER3 co-amplification were detectable as homogenous and broad cluster-amplification of HER3 (HER3ba). These tumors were all assigned to the first two reclassified subgroups of LPS (WDLS/DDLS). In contrast, among cases showing single-occurring or small area HER3 cluster amplifications (HER3f), only eight of the total ten cases were MDM2-coamplified, although these eight samples referred also to the first subgroups of reclassified LPS (WDLS/DDLS) while the other two without MDM2 co-amplification were one of the pleomorphic subgroup and one lipoma. By immunohistochemistry, HER3 expression could be observed not as homogeneously positive as the amplification of HER3. That is most likely based on low specificity of the tested and used antibodies and will be a subject of a subsequent research project.

Biological background must be recognized in more detail to clarify why in some tumors HER3 was cluster amplified in large areas and in others only in single cells: ring chromosomes are frequently present in various neoplasms, especially in mesenchymal tumors as liposarcomas, which -depending on the type- show ring chromosomes in more than 70% of cases [18, 31]. Similarly, ring chromosomes are present in well differentiated and dedifferentiated liposarcomas but also in undifferentiated pleomorphic liposarcoma in 15–25% of cases. Even in lipoma ring chromosomes could be detected [32]. The ring and giant marker chromosomes, which are found in 90% of WDLS/DDLS, are very unstable, so that ring chromosomes break during tumor progression [33]. Jagosky et al. describes genetic instabilities of ring chromosomes as well in dedifferentiated liposarcoma, harboring (co-)amplifications of MDM2 in 75%, CDK4 in 65% and HMGA2 in 29% and Ingham et al. additionally CDKN2A amplification in 23% of cases [9, 18]. These broken rings then either reconnect or transform into giant marker chromosomes that attach to telomeres or telomere sequences of other chromosomes. This results in wide variability in the size and content of ring and giant marker chromosomes in different cells of the same lesion and in the number of rings located within a tumor cell or even within the entire lesion [34]. This is most likely the basis of the described findings of tumors showing MDM2 amplifications not consistently harboring HER3 co-amplifications. Furthermore, this genetic background could explain why not all tumors were HER3-amplified homogeneously but revealed focal cluster amplifications. Nevertheless, genes that serve cell proliferation lead in case of amplification in tumor cells to a selection advantage of the latter. Since MDM2 inhibits DNA repair mechanisms, it has such a selection advantage [35]. Further studies revealed decreased overall survival in DDLS in cases of CDK4 and HMGA2 co-amplifications [18]. HER3, on the other hand, tends might show a selection advantage by its nature as a receptor tyrosine kinase, but is only coamplified by proximity [10]. In different publications, co-amplifications were investigated as well, in some using NGS technology and whole exome or target enrichment sequencing [10, 36]. The frequency of detected HER3-amplification was much lower than in our study, revealing only up to 7% and 18% in all RTKs [10]. By NGS, as tumor tissue is homogenized and DNA is extracted, a detection of an amplification of genes need to be broad and homogeneous to be detected as a valid amplification. HER3 showed in our study and also by Asano et al. using FISH-technology an inhomogeneous amplification pattern [10]. Therefore, higher rates of HER3 amplification can be detected using FISH technology as it discovers low abundant and also low numeric amplifications.

Some authors investigated HER3 amplification in inhomogeneous tumors harboring dedifferentiated areas and well differentiated areas [10]. As we constructed a TMA for FISH analysis, we selected homogenous tumor tissue reflecting the original diagnosis therefore not suitable for this question. But we can confirm higher rates of HER3 amplifications in dedifferentiated LPS, thus suggesting, as Asano et al. hypothesized, being associated with progression from WDLS to DDLS.

**Fig. 5:**
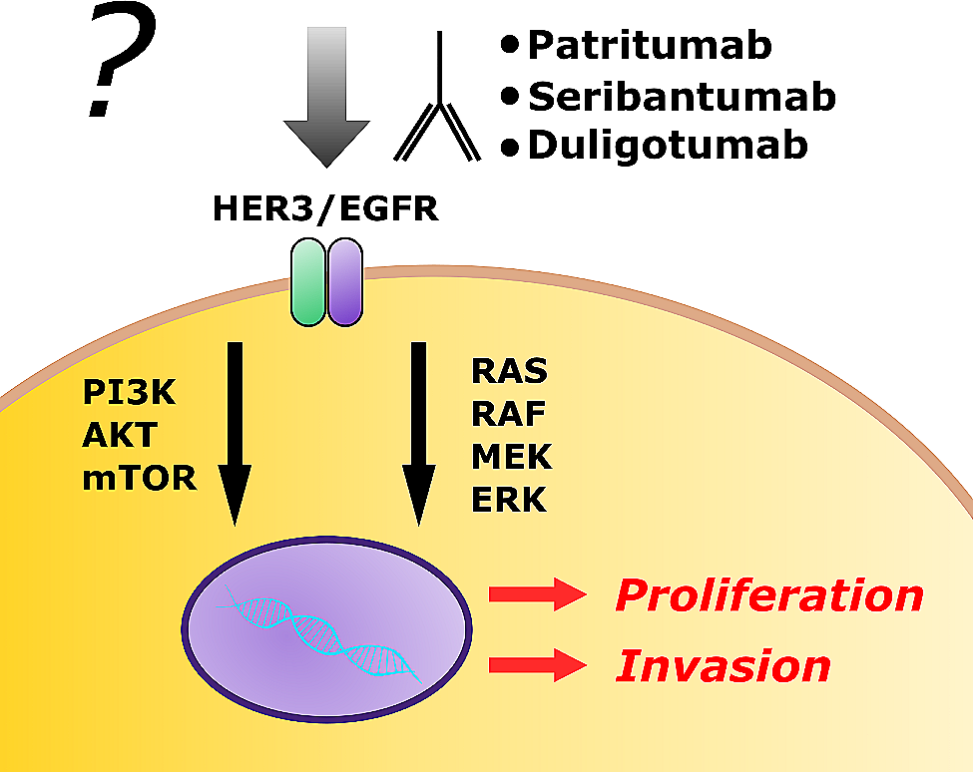
A possible approach for targeted therapy in liposarcomas via HER3. Human monoclonal antibodies such as patritumab, seribantumab or duligotumab inhibit as pan-RTK-inhibitors the activation of the HER3/EGFRx receptor. Via PI3K-AKT-mTOR or RAS-RAF-MEK-ERK signaling cascade, tumor proliferation and invasion could be reduced by receptor inhibition

HER3, as a member of the tyrosine kinase group of human epidermal growth receptors, plays a critical role in cell-survival and drug resistance in malignancies. Even showing low kinase activity by itself, it could serve as a driver of cell proliferation if mutated. Building heterodimers with other receptor tyrosine kinases as of the Her family (EGFR, HER2, HER4) cells are able to escape targeted therapies and chemotherapy (Fig. 5) [37]. Targeting HER3 itself or different RTKs simultaneously by panHer inhibitors might support systemic therapy of unresectable liposarcomas harboring HER3 amplifications (Fig. 5) [38]. Therefore, blocking was determined as putative targeted therapy [27] using monoclonal antibodies as patritumab, seribantumab, duligotumab or lumretuzumab (targeting both, EGFR1 and HER3) [25, 39]. Some antibody-drug conjugate are considered as therapeutics as well, as in trials e.g. for prostate cancer or in non-small-cell lung cancer [25, 40]. Next to monoclonal antibodies, specific inhibitors are under investigation in clinical trials as for breast cancer [41]. Described by Ingham in 2023, a trial using multi-tyrosine-kinase-inhibitor sitravatinib showed a “meaningful disease control” [9]. Pan-RTK-inihibitors are established in clinical treatment but to our knowledge not yet well correlated to HER3 amplifications. Specific HER3-inhibitors had not yet been approved or established in clinical practice.

In future, next to the detection of HER3 amplification, Heregulin, a ligand to HER3, discussed to serve as a putative biomarker to predict targeted therapy to HER3 [39], could possibly analyzed subsequently by immunohistochemistry. Immunohistochemistry of HER3 directly showed some correlation but by used antibodies not a strong, reliant result in our samples as HER2 (ERBB2) generally correlating well to HER2 amplification in cancer as a very established and standardized staining [42]. In some carcinoma, the staining could be better established in recent years as in colorectal carcinoma [43].

In older cases of the archive, at that time not yet classified by FISH as a standard, many cases needed to be reclassified concerning subgroups. Focusing on the subgroup of myxoid liposarcomas demonstrating MDM2 amplification cases were stratified as dedifferentiated liposarcomas as myxoid appearance of MDM2 amplified liposarcomas, especially DDLS, was also described in literature [44–47]. Furthermore in MLS, FUS-DDIT3 rearrangements represent not the only genetic alteration but most frequent next to other translocations, e.g. EWSR1-DDIT3 which might not be detectable by the used break apart probes in FISH [48]. While FISH analyses are cost effective and rapidly done, fusion panel analysis using NGS (next generation sequencing) covers better possible fusions but a cost intensive and time consuming [49]. The reclassification performed by FISH and IHC reclassified a total of 30% of the tumors. Kashima et al. demonstrated similar results using FISH in archived cases leading to reclassification of cases [50, 51]. Similar results were found in studies comparing histomorphology retrospectively adding fluorescence in-situ-hybridization leading to reassignment of cases [52, 53]. Concerning consequences of reclassification for patients are under discussion with the ethical board due to anonymization of samples.

Limitations to this study are on firsthand relatively low numbers of subtypes of liposarcomas and therefore low representation of this tumor entity in general and might be validated on higher numbers of samples. However, even including a rather low sample size, we already could show a high number of cases presenting HER3 amplifications. Nevertheless, presented results are concordant throughout the samples and show significant correlations. Furthermore, in subsequent studies it should be analyzed in functional assays on cell culture and liposarcoma cell lines harboring HER3 amplifications. Additionally, in clinical trials targeting HER3 or using multi-kinase inhibitors the amplification status could serve to search for correlations in respect to response rates.

## Conclusions

In summary, we could demonstrate that immunohistochemistry and fluorescence in-situ-hybridization are well established as well necessary for the diagnosis and stratification of subtypes of liposarcoma. Next, we could demonstrate a correlation between HER3 and MDM2 amplification, supporting the hypothesis that HER3 is coamplified in a major part of some subgroups of liposarcomas due to its spatial proximity to MDM2. As HER3 serves in trials as a target structure in personalized medicine in malignant tumors, HER3 might serve as a target structure and stratifying tool in unresectable or metastasized liposarcomas showing a HER3 amplification.

## Data Availability

The datasets used during the current study are available from the corresponding author on reasonable request.

## Declarations

### Inclusion of identifiable human data

No potentially identifiable human images or data is presented in this study.

## Abbreviations

FISH: Fluorescence in situ hybridization
IHC: Immunohistochemistry
STS: Soft Tissue Sarcomas
LPS: Liposarcomas
ALT: Atypical Lipomatous Tumors
WDLS: Well-Differentiated LPS
DDLS: Dedifferentiated LPS
MLS: Myxoid or round cell LPS
PLS: Pleomorphic LPS
CDKN2A: Cyclin-Dependent Kinase inhibitor 2 A
LGR5: Leucine-rich repeat-containing G protein-coupled Receptor 5
RTK: Receptor Tyrosine Kinases
IRS: Immunoreactivity score

## References

[1] Ferrari A, Sultan I, Huang TT (2011). Soft tissue sarcoma across the age spectrum: a population-based study from the Surveillance Epidemiology and End results database. Pediatr Blood Cancer.

[2] Stiller CA, Trama A, Serraino D (2013). Descriptive epidemiology of sarcomas in Europe: report from the RARECARE project. Eur J Cancer.

[3] Yang Z, Zheng R, Zhang S (2019). Incidence, distribution of histological subtypes and primary sites of soft tissue sarcoma in China. Cancer Biol Med.

[4] WHO. (2020) Soft tissue and bone tumours, 5th edition. World Health Organization classification of tumours series, vol. 3, 5th ed. World Health Organization; International Agency for Research on Cancer, Geneva, Lyon.

[5] Creytens D (2020). What’s new in adipocytic neoplasia?. Virchows Arch.

[6] Creytens D, Folpe AL, Koelsche C (2021). Myxoid pleomorphic liposarcoma-a clinicopathologic, immunohistochemical, molecular genetic and epigenetic study of 12 cases, suggesting a possible relationship with conventional pleomorphic liposarcoma. Mod Pathol.

[7] Pilotti S, Della Torre G, Mezzelani A (2000). The expression of MDM2/CDK4 gene product in the differential diagnosis of well differentiated liposarcoma and large deep-seated lipoma. Br J Cancer.

[8] Thway K (2019). Well-differentiated liposarcoma and dedifferentiated liposarcoma: an updated review. Semin Diagn Pathol.

[9] Ingham M, Lee S, van Tine BA (2023). A single-arm phase II trial of Sitravatinib in Advanced Well-Differentiated/Dedifferentiated Liposarcoma. Clin Cancer Res.

[10] Asano N, Yoshida A, Mitani S (2017). Frequent amplification of receptor tyrosine kinase genes in welldifferentiated/ dedifferentiated liposarcoma. Oncotarget.

[11] Downs-Kelly E, Goldblum JR, Patel RM (2008). The utility of fluorescence in situ hybridization (FISH) in the diagnosis of myxoid soft tissue neoplasms. Am J Surg Pathol.

[12] Antonescu CR, Elahi A, Humphrey M (2000). Specificity of TLS-CHOP rearrangement for Classic Myxoid/Round Cell Liposarcoma. J Mol Diagn.

[13] Schneider-Stock R, Walter H, Radig K (1998). MDM2 amplification and loss of heterozygosity at rb and p53 genes: no simultaneous alterations in the oncogenesis of liposarcomas. J Cancer Res Clin Oncol.

[14] Thway K (2022). What’s new in adipocytic neoplasia?. Histopathology.

[15] Jones RL, Fisher C, Al-Muderis O (2005). Differential sensitivity of liposarcoma subtypes to chemotherapy. Eur J Cancer.

[16] Smith CA, Martinez SR, Tseng WH (2012). Predicting survival for well-differentiated liposarcoma: the importance of tumor location. J Surg Res.

[17] Henricks WH, Chu YC, Goldblum JR et al. Dedifferentiated Liposarcoma: a clinicopathological analysis of 155 cases with a proposal for an expanded definition of Dedifferentiation. American Journal of Surgical Pathology. 1997:31. 10.1097/00000478-199703000-00002 9060596

[18] Jagosky MH, Anderson CJ, Symanowski JT (2023). Genomic alterations and clinical outcomes in patients with dedifferentiated liposarcoma. Cancer Med.

[19] Haniball J, Sumathi VP, Kindblom L-G (2011). Prognostic factors and metastatic patterns in primary myxoid/round-cell liposarcoma. Sarcoma.

[20] Moreau L-C, Turcotte R, Ferguson P (2012). Myxoid\round cell liposarcoma (MRCLS) revisited: an analysis of 418 primarily managed cases. Ann Surg Oncol.

[21] Ghadimi MP, Liu P, Peng T (2011). Pleomorphic liposarcoma: clinical observations and molecular variables. Cancer.

[22] Wan L, Tu C, Qi L (2021). Survivorship and prognostic factors for pleomorphic liposarcoma: a population-based study. J Orthop Surg Res.

[23] Nassif NA, Tseng W, Borges C et al. Recent advances in the management of liposarcoma. F1000Res 2016;5:2907. 10.12688/f1000research.10050.1. 10.12688/f1000research.10050.1 PMC5224678 28105325

[24] Du Z, Lovly CM (2018). Mechanisms of receptor tyrosine kinase activation in cancer. Mol Cancer.

[25] Trinder A, Ding K, Zhang J (2024). The therapeutic significance of HER3 in non-small cell Lung Cancer (NSCLC): a review study. Curr Med Chem.

[26] Cherifi F, Da Silva A, Martins-Branco D, et al. Pharmacokinetics and pharmacodynamics of antibody-drug conjugates for the treatment of patients with breast cancer. Expert Opin Drug Metab Toxicol. 2024;1–15. 10.1080/17425255.2024.2302460. 10.1080/17425255.2024.2302460 38214896

[27] Gala K, Chandarlapaty S (2014). Molecular pathways: HER3 targeted therapy. Clin Cancer Res.

[28] Thomas G, Chardès T, Gaborit N (2014). HER3 as biomarker and therapeutic target in pancreatic cancer: new insights in pertuzumab therapy in preclinical models. Oncotarget.

[29] Dei Tos AP (2014). Liposarcomas: diagnostic pitfalls and new insights. Histopathology.

[30] Gibas Z, Miettinen M, Limon J (1995). Cytogenetic and immunohistochemical profile of myxoid liposarcoma. Am J Clin Pathol.

[31] Gebhart E (2008). Ring chromosomes in human neoplasias. Cytogenet Genome Res.

[32] Italiano A, Cardot N, Dupré F (2007). Gains and complex rearrangements of the 12q13-15 chromosomal region in ordinary lipomas: the missing link between lipomas and liposarcomas?. Int J Cancer.

[33] Pedeutour F, Suijkerbuijk RF, Forus A (1994). Complex composition and co-amplification of SAS and MDM2 in ring and giant rod marker chromosomes in well-differentiated liposarcoma. Genes Chromosom Cancer.

[34] Stephenson CF, Berger CS, Leong SP (1992). Analysis of a giant marker chromosome in a well-differentiated liposarcoma using cytogenetics and fluorescence in situ hybridization. Cancer Genet Cytogenet.

[35] Oliner JD, Saiki AY, Caenepeel S. The role of MDM2 amplification and overexpression in Tumorigenesis. Cold Spring Harb Perspect Med. 2016;6. 10.1101/cshperspect.a026336. 10.1101/cshperspect.a026336 PMC4888815 27194168

[36] Kanojia D, Nagata Y, Garg M (2015). Genomic landscape of liposarcoma. Oncotarget.

[37] Lyu H, Han A, Polsdofer E (2018). Understanding the biology of HER3 receptor as a therapeutic target in human cancer. Acta Pharm Sinica B.

[38] Gaborit N, Lindzen M, Yarden Y (2016). Emerging anti-cancer antibodies and combination therapies targeting HER3/ERBB3. Hum Vaccines Immunotherapeutics.

[39] Kawakami H, Yonesaka K (2016). HER3 and its Ligand, Heregulin, as targets for Cancer Therapy. PRA.

[40] Gil V, Miranda S, Riisnaes R (2021). HER3 is an actionable target in advanced prostate Cancer. Cancer Res.

[41] Johnston S, Basik M, Hegg R (2016). Inhibition of EGFR, HER2, and HER3 signaling with AZD8931 in combination with anastrozole as an anticancer approach: phase II randomized study in women with endocrine-therapy-naïve advanced breast cancer. Breast Cancer Res Treat.

[42] Jimenez RE, Wallis T, Tabasczka P (2000). Determination of Her-2/Neu status in breast carcinoma: comparative analysis of immunohistochemistry and fluorescent in situ hybridization. Mod Pathol.

[43] Capone E, Tryggvason T, Cela I (2023). HER-3 surface expression increases in advanced colorectal cancer representing a potential therapeutic target. Cell Death Discov.

[44] Mentzel T, Fletcher CD (1997). Dedifferentiated myxoid liposarcoma: a clinicopathological study suggesting a closer relationship between myxoid and well-differentiated liposarcoma. Histopathology.

[45] de Vreeze RSA, de Jong D, Koops W (2011). Oncogenesis and classification of mixed-type liposarcoma: a radiological, histopathological and molecular biological analysis. Int J Cancer.

[46] Boland JM, Colby TV, Folpe AL (2012). Liposarcomas of the mediastinum and thorax: a clinicopathologic and molecular cytogenetic study of 24 cases, emphasizing unusual and diverse histologic features. Am J Surg Pathol.

[47] Sioletic S, Dal Cin P, Fletcher CDM (2013). Well-differentiated and dedifferentiated liposarcomas with prominent myxoid stroma: analysis of 56 cases. Histopathology.

[48] Bode-Lesniewska B, Frigerio S, Exner U (2007). Relevance of translocation type in myxoid liposarcoma and identification of a novel EWSR1-DDIT3 fusion. Genes Chromosom Cancer.

[49] Scheipl S, Brcic I, Moser T (2021). Molecular profiling of soft-tissue sarcomas with FoundationOne® Heme identifies potential targets for sarcoma therapy: a single-centre experience. Ther Adv Med Oncol.

[50] Kashima T, Halai D, Ye H (2012). Sensitivity of MDM2 amplification and unexpected multiple faint alphoid 12 (alpha 12 satellite sequences) signals in atypical lipomatous tumor. Mod Pathol.

[51] Asif A, Mushtaq S, Hassan U (2018). Fluorescence in situ hybridization (FISH) for Differential diagnosis of soft tissue sarcomas. Asian Pac J Cancer Prev.

[52] Vroobel K, Gonzalez D, Wren D (2016). Ancillary molecular analysis in the diagnosis of soft tissue tumours: reassessment of its utility at a specialist centre. J Clin Pathol.

[53] Vargas AC, Joy C, Cheah AL (2022). Lessons learnt from MDM2 fluorescence in-situ hybridisation analysis of 439 mature lipomatous lesions with an emphasis on atypical lipomatous tumour/well-differentiated liposarcoma lacking cytological atypia. Histopathology.

